# Supplementary material for: Septum site placement in Mycobacteria – identification and characterisation of mycobacterial homologues of Escherichia coli MinD
Source: Microbiology (Reading). 2023 Aug 1;169(8):001359. doi: 10.1099/mic.0.001359 (PMC10482377; doi:10.1099/mic.0.001359)
Supplement: Supplementary material 1 [file mic-169-1359-s001.pdf]

Subject / Query	*Ec* MinD (270 aa)	Rv1708 (318 aa)	Rv3660c (350 aa)	MSMEG_3743 (297 aa)	MSMEG_6171 (413 aa)
***Ec* MinD (270 aa)**	100	Aligned 2-195 Identity 24% (50/212) Positives 48 % (103/212)	Aligned 4-150 Identity 23% (35/149) Positives 41 % (62/149)	Aligned 2-195 Identity 25% (52/212) Positives 48 % (102/212)	Aligned 4-150 Identity 23% (34/147) Positives 39 % (58/147)
**Rv1708 (318 aa)**	Aligned 64-261 Identity 24% (50/212) Positives 48 % (103/212)	100	Aligned 66-103 Identity 34% (13/38) Positives 52 % (20/38)	Aligned 21-318 Identity 85% (253/298) Positives 91% (272/298)	Aligned 66-103 Identity 39% (15//38) Positives 47% (18/38)
**Rv3660c (350 aa)**	Aligned 117-255 Identity 23% (33/146) Positives 41 % (61/146)	Aligned 267-342 Identity 25% (22/88) Positives 40 % (35/88)	100	Aligned 117-150 Identity 37% (14/38) Positives 55% (21/38)	Aligned 1-138 Identity 60% (203/338) Positives 71% (241/338)
**MSMEG_3743 (297 aa)**	Aligned 43-240 Identity 25% (52/212) Positives 48 % (102/146)	Aligned 2-297 Identity 81% (253/298) Positives 91 % (272/298)	Aligned 45-238 Identity 26% (23/207) Positives 36 % (75/207)	100	Aligned 40-82 Identity 40% (17/43) Positives 48% (21/43)
**MSMEG_6171 (413 aa)**	Aligned 183-296 Identity 23% (27/119) Positives 40% (48/119)	Aligned 183-216 Identity 39% (15/38) Positives 47 % (18/38)	Aligned 72-404 Identity 60% (203/338) Positives 71% (241/338)	Aligned 183-216 Identity 39% (15/38) Positives 50% (19/38)	100

Figure S1: **Checkerboard showing sequence conservation of *E. coli* MinD and its mycobacterial homologues Rv1708/MSMEG_3743 and Rv3660c/MSMEG_6171**

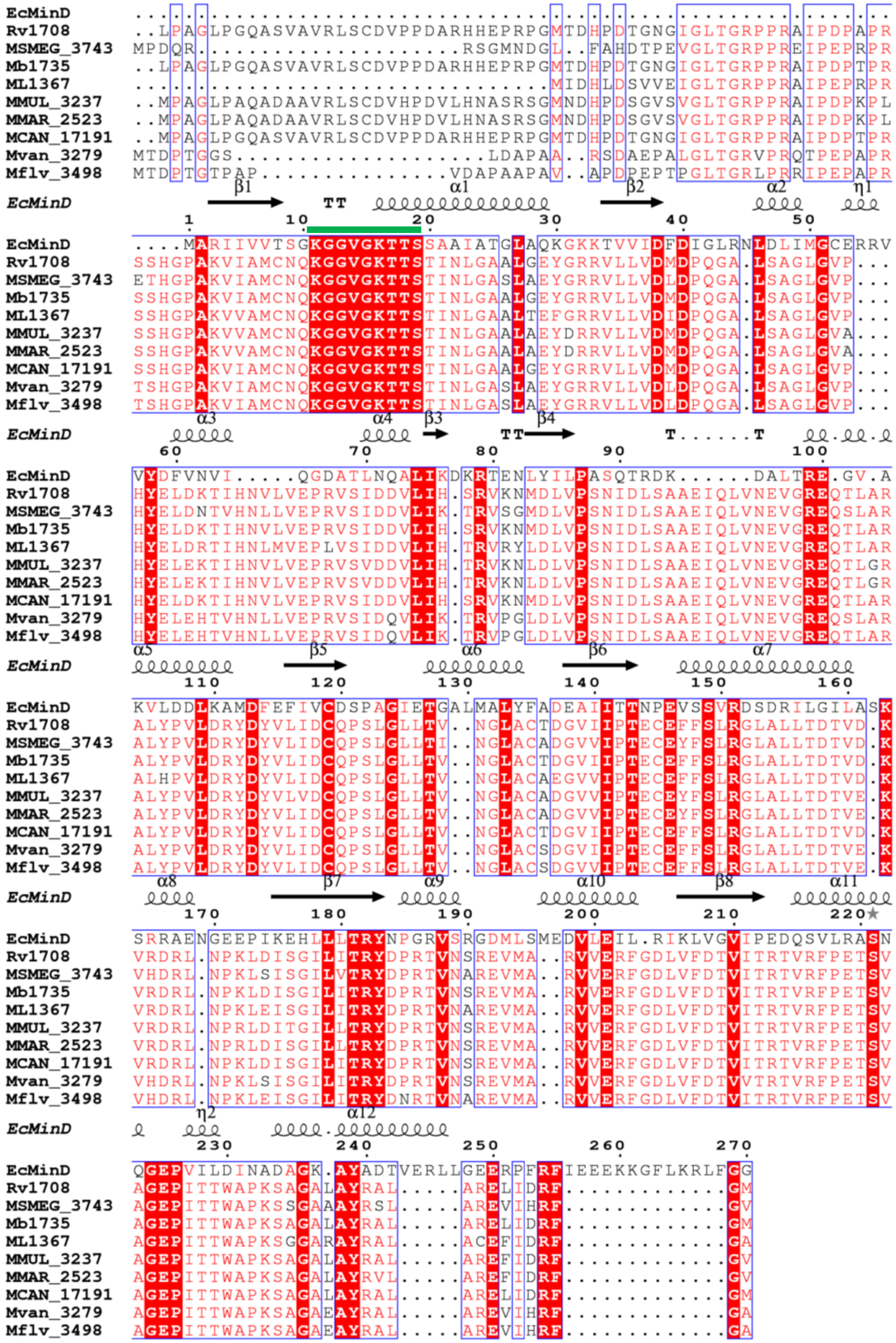

Figure S2: **Conservation of MSMEG_3743/Rv1708 sequences across mycobacteria.** The green line shows conservation of the Deviant Walker A motif. Mmul-*Mycobacterium ulcerans,* Mmar-*Mycobacterium marinum,* Ml-*Mycobacterium leprae,* Mcan-*Mycobacterium canetti,* Rv-*Mycobacterium tuberculosis,* Mb-*Mycobacterium bovis,* MSMEG-*Mycobacterium smegmatis,* Mvan-*Mycobacterium vanbaalenii,* Mflv-*Mycobacterium gilvum*.

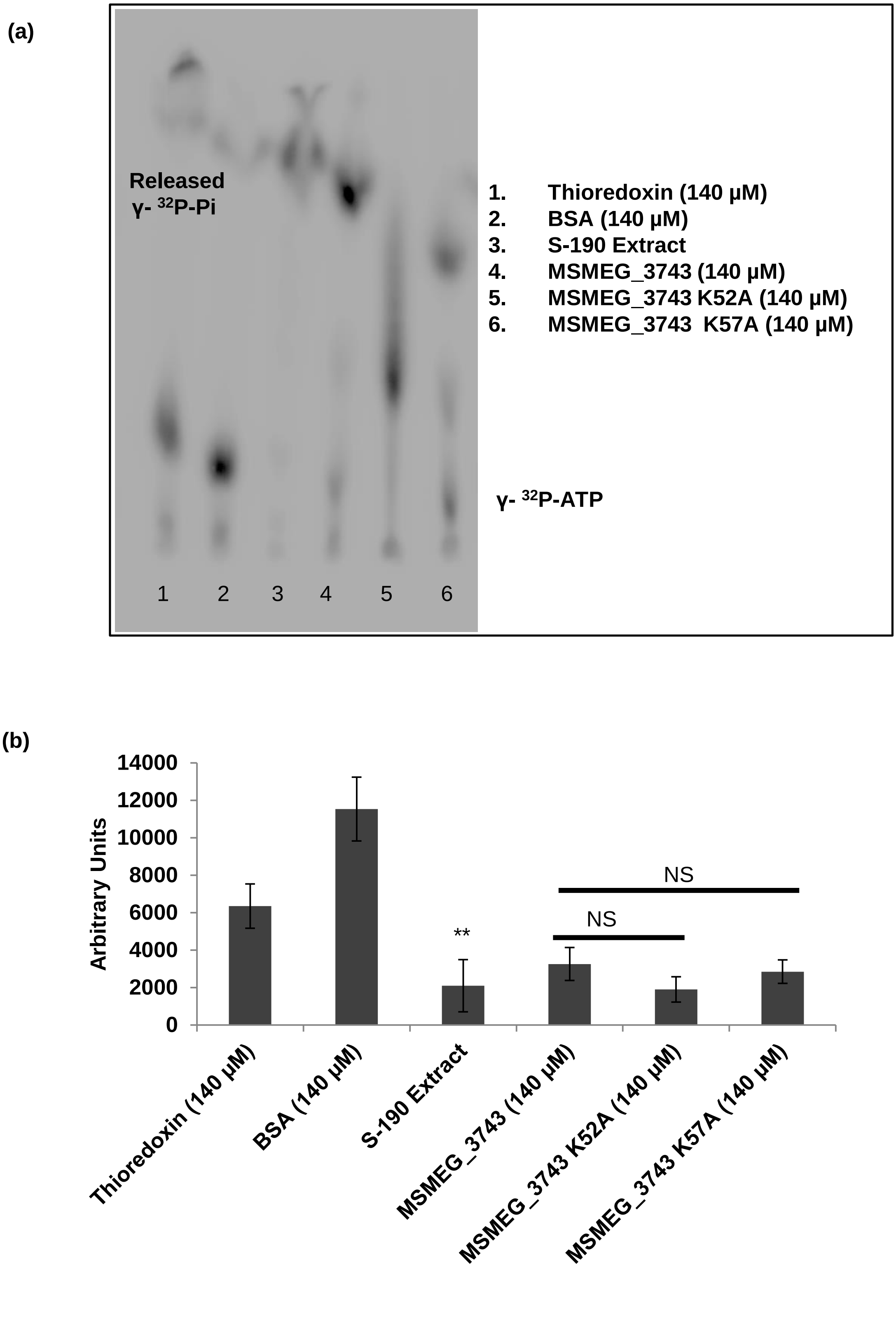

Figure S3: **ATPase activity of Lysine mutants of MSMEG_3743.** (a) Radiograph of γ-^{32}P-Pi release from hydrolysis of radiolabelled ATP. (b) Densitometric quantitation of unhydrolysed γ-^{32}P-ATP from each lane of the radiograph. Error bars represent SD; ** P<0.01, T-test done wrt. BSA. NS – Not Significant.

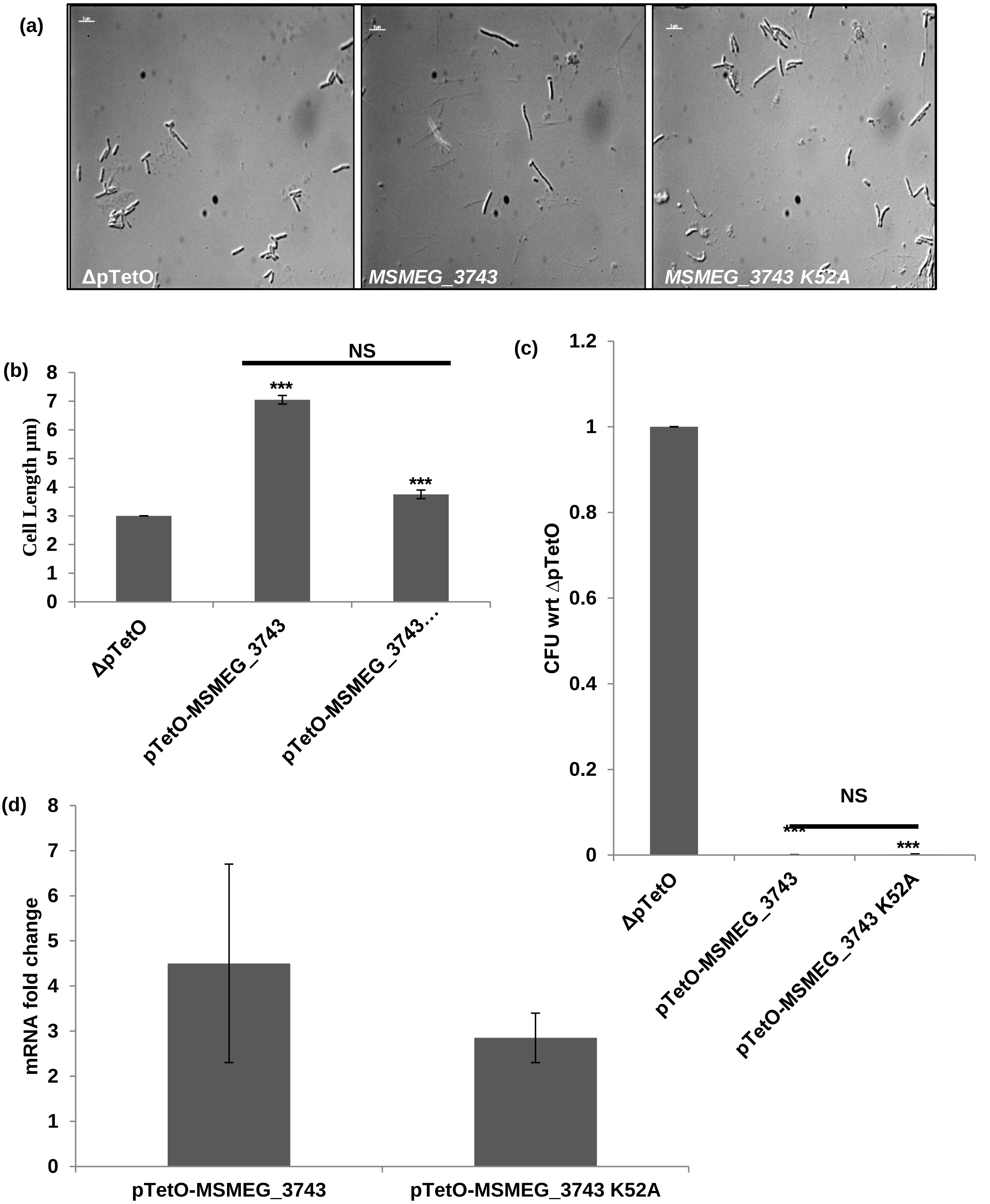

Figure S4: **Effect of overexpression of *MSMEG_3743 K52A* in *M. smegmatis*.** (a) Representative DIC images of overexpression strains; Scale bar 2 µm (b) Average cell lengths of overexpression strains (µm) Error bars represent SEM. (c) CFU counts, represented as a ratio wrt ΔpTetO (d) Transcript levels of *MSMEG_3743* & *MSMEG_3743* K52A following overexpression, wrt *Ms sigA.* *** P<0.001, T-test performed wrt ΔpTetO ; NS - Not Significant.

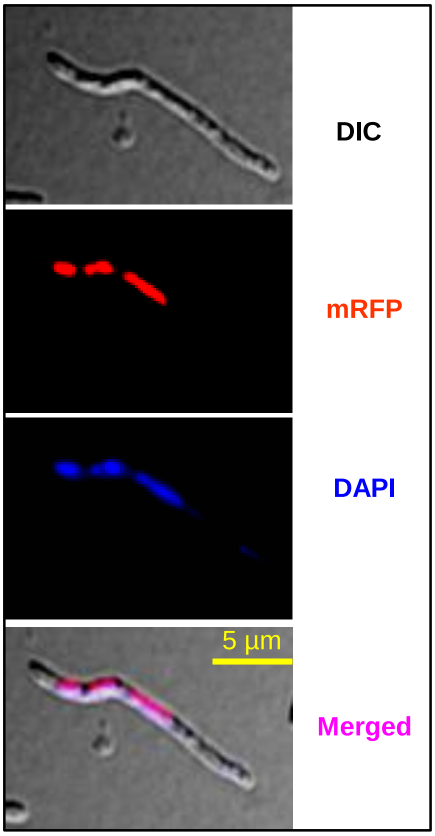

Figure S5: **Interaction of MSMEG_3743 with the bacterial chromosome.** Representative micrographs of *M. smegmatis* expressing *MSMEG_3743-mRFP* stained with DAPI; Scale bar 5 µm.

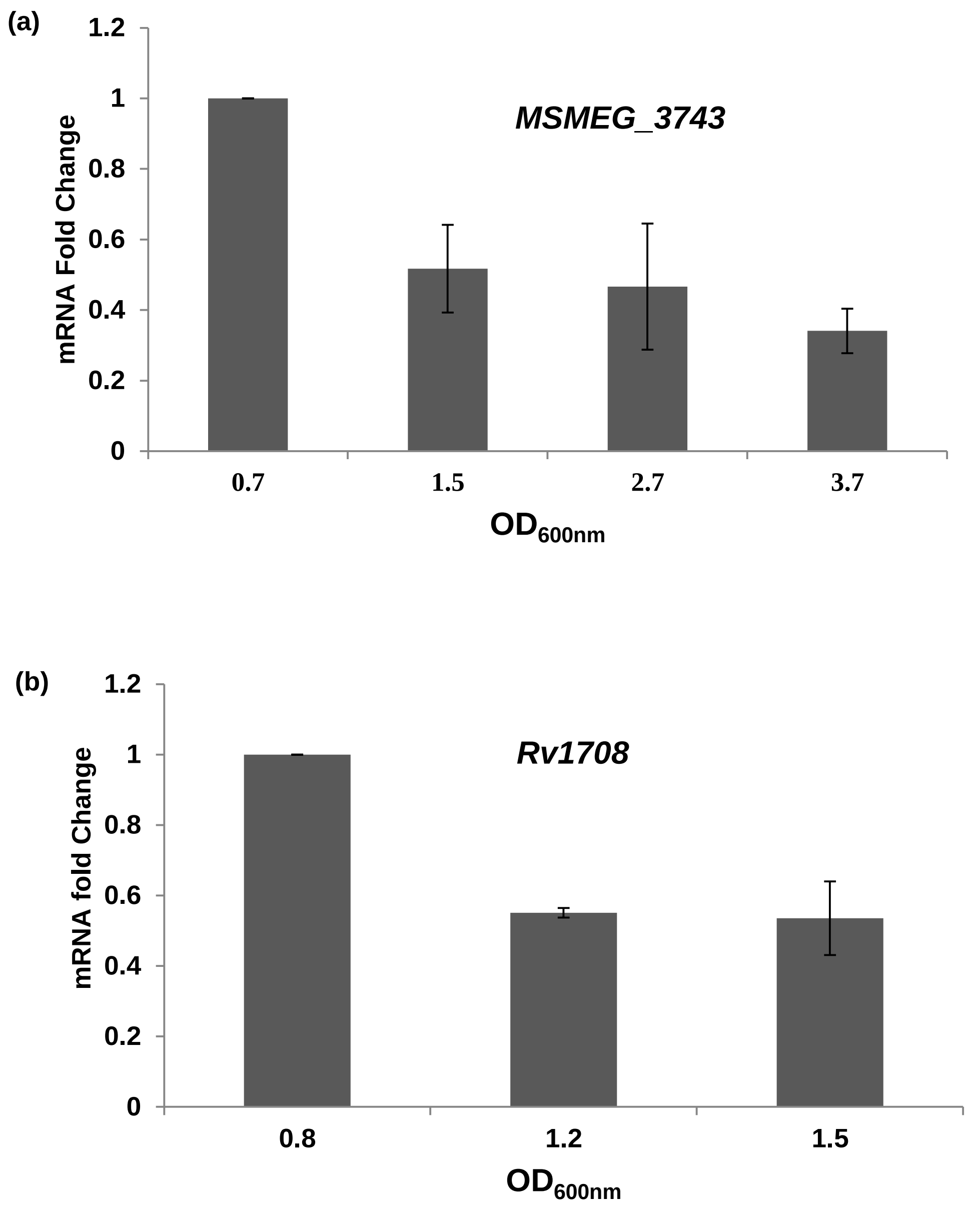

Figure S6: **Growth phase dependent gene expression of mycobacterial *minD* homologues.** Transcript levels of *MSMEG_3743* in *M. smegmatis* (a) and *Rv1708* in *M.tb* (b) at different phases of growth relative to their levels at mid-log phase. Error bars represent SD.

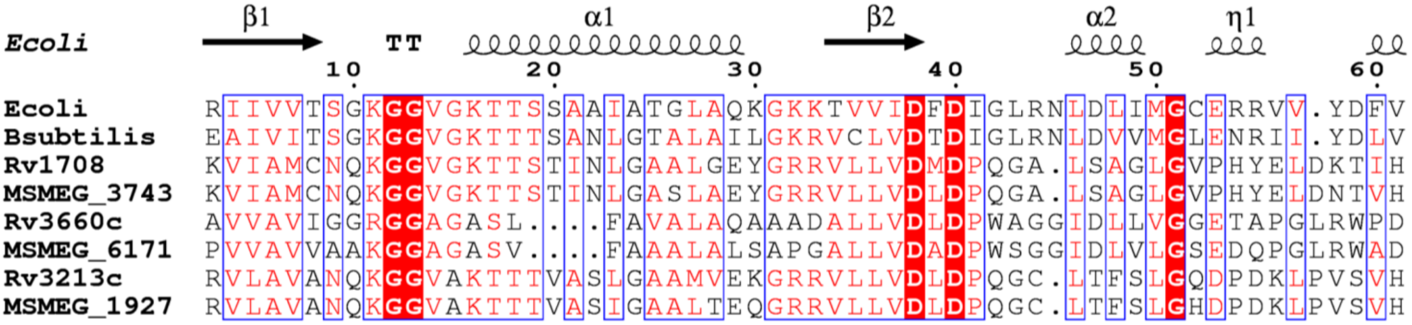

Figure S7: Snippet of the sequence alignment containing the deviant Walker A motif (see figure S2) in *E.coli* MinD and its predicted homologues. The two Glycine residues can be clearly seen to be conserved in all sequences.

(a)

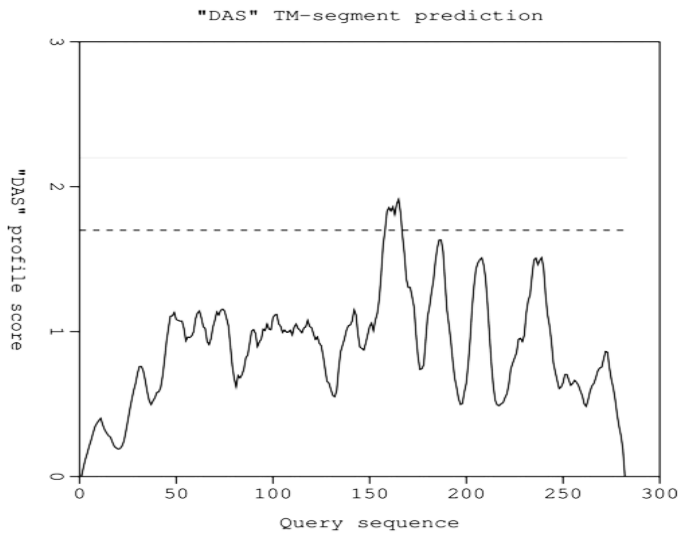

(b)

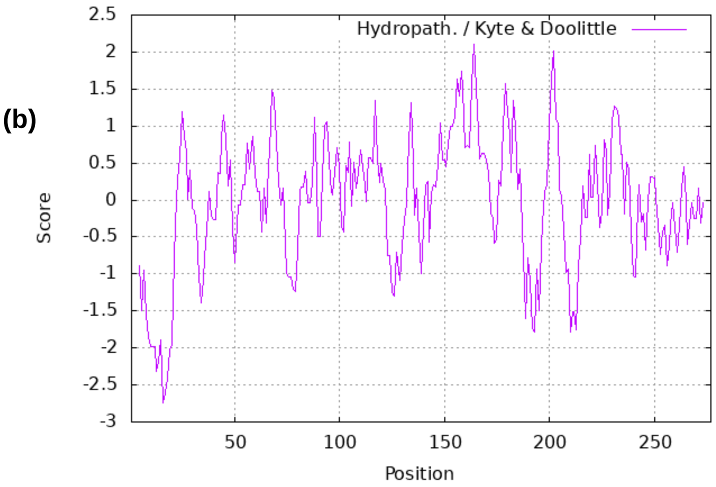

Figure S8:. Transmembrane prediction (a), and Hydrophobicity (b) analyses of MSMEG_3743

Primer Name	Description	Sequence (5'-3')
pTrc99A-*MSMEG_3743*-FP	FP (Forward Primer) for cloning *MSMEG_3743* in pTrc99A	ATGCAGAATTCATGCCTGACCAGCGGCGG
pTrc99A-*MSMEG_3743*-RP	RP (Reverse Primer) for cloning *MSMEG_3743* in pTrc99A	TGCATGGATCCTCACACGCCGAACCGGTG
pTrc99A- *MSMEG _6171*-FP	FP for cloning *Ms_6171* in pTrc99A	ATGCAGAATTCTTGCCCGTCATTGCCGGGC
pTrc99A- *MSMEG _6171*-RP	RP for cloning *Ms_6171* in pTrc99A	TGCATAAGCTTTCACGCCGCCTCCCGCAC
pTrc99A- *E. coli minD*-FP	FP for cloning *E. coli minD* in pTrc99A	ATGCAGAATTCATGGCACGCATTATTGTTG
pTrc99A- *E. coli minD*-RP	RP for cloning *E. coli minD* in pTrc99A	TGCATGGATCCTTATCCTCCGAACAAGCG
pTrc99A-*Rv1708*-FP	FP for cloning *Rv1708* in pTrc99A	ATGCAGAATTCTTGCCTGCGGGTCTCCCG
pTrc99A-*Rv1708*-RP	FP for cloning *Rv1708* in pTrc99A	TGCATAAGCTTTCACATGCCAAATCGGTCGA
pTrc99A-*Rv3660c*-FP	FP for cloning *Rv3660c* in pTrc99A	ATGCAGAATTCATGCTGACCGATCCGGGG
pTrc99A-*Rv3660c*-RP	RP for cloning *Rv3660c* in pTrc99A	TGCATGGATCCTCATGCCGCCCTACCGTG
pTetO-*MSMEG_3743*-FP	FP for cloning *MSMEG_3743* in pTetO	ATGCACATATGATGCCTGACCAGCGGCGG
pTetO-*MSMEG_3743*-RP	RP for cloning *MSMEG_3743* in pTetO	TGCATAAGCTTTCACACGCCGAACCGGTG
pTetO- *MSMEG _6171*-FP	FP for cloning *MSMEG _6171* in pTetO	ATGCACATATGTTGCCCGTCATTGCCGGGC
pTetO- *MSMEG _6*171-RP	RP for cloning *MSMEG _6171* in pTetO	TGCATAAGCTTTCACGCCGCCTCCCGCAC
pTetO- *Msm scpA*-FP	FP for cloning *Msm scpA* in pTetO	ATGCACATATGGTGAACGACGACGTGCGT
pTetO- *Msm scpA*-RP	RP for cloning *Msm scpA* in pTetO	TGCATAAGCTTCTATTCTTCCGCATCGGC
pTetO-*Msm scpB*-FP	FP for cloning *Msm scpB* in pTetO	ATGCACATATGATGACTGACGAGACCTCC
pTetO- *Msm scpB*-RP	RP for cloning *Msm scpB* in pTetO	TGCATAAGCTTTCAATCCTTGTCCACGTC
pTetO- *Msm parB*-FP	FP for cloning *Msm parB* in pTetO	ATGCACATATGATGAATCAGCCGGCACGC
pTetO- *Msm parB*-RP	RP for cloning *Msm parB* in pTetO	TGCATAAGCTTTTACTCGTTCTGGGCGCTC
pSCW54-*MSMEG_3743*-FP	FP for cloning *MSMEG_3743* in pSCW54	ATGCACATATGATGCCTGACCAGCGGCGG
pSCW54-*MSMEG_3743*-RP	RP for cloning *MSMEG_3743* in pSCW54	TGCATTTAATTAATCACACCGCCGAACCGGTG
pSCW54-6x His-*MSMEG_3743*-FP	FP for cloning N-terminal His-tagged *MSMEG_3743* in pSCW54	ATGCACATATGATGCACCACCACCACCAC CACCCTGACCAGCGGCGGTCG
pSCW54-*MSMEG_3743*-6x His- RP	FP for cloning C-terminal His-tagged *MSMEG_3743* in pSCW54	TGCATTTAATTAATCAGTGGTGGTGGTGGTG GTGCACGCCGAACCGGTG
pTetO-*MSMEG_3743*-NTL-FP	FP for cloning N-terminal mRFP fusion with *MSMEG_3743* in pSCW54	ATGCAAAGCTTGGAGGAGGAGGAGGAATG CCTGACCAGCGGCGG
pTetO-*MSMEG_3743*-CTL-RP	RP for cloning N-terminal mRFP fusion with *MSMEG_3743* in pSCW54	TGCATCATATGTCCTCCTCCTCCTCCCACGCCG AACCGGTGGAT
MSMEG_3743-RT-FP	FP for RT PCR of *MSMEG_3743*	TCGGCAAGACCACGTCGA

MSMEG_3743-RT-RP	RP for RT PCR of *MSMEG_3743*	TCAGCACGTCGTCGATGG
MSMEG _6171-RT-FP	FP for RT PCR of *MSMEG _6171*	AGTCGTAAGGCCTGGCTG
MSMEG _6171-RT-RP	RP for RT PCR of *MSMEG _6171*	GCCACAAGATCGGTGTCC
Msm sigA-RT-FP	FP for RT PCR of *Msm sigA*	GCCAGCTCGGTGACTTCA
Msm sigA-RT-RP	RP for RT PCR of *Msm sigA*	CGTGACGCCGTAGACCTG
Msm parB-RT-FP	FP for RT PCR of *Msm parB*	CGAGTTCGGTCTCATGCAG
Msm parB-RT-RP	RP for RT PCR of *Msm parB*	GTTCAACTGGACGCGGTG
Rv1708-RT-FP	FP for RT PCR of *Rv1708*	ATGGATCCGCAAGGAGCG
Rv1708-RT-RP	RP for RT PCR of *Rv1708*	ACCCACCTCGTTGACCAG
Mtb. sigA-RT-FP	FP for RT PCR of *Mtb. sigA*	AAACAGATCGGCAAGGTAGC
Mtb. sigA-RT-RP	RP for RT PCR of *Mtb. sigA*	TCCAGCGATGGTTTTTCG
pET22b-*MSMEG_3743*-FP	FP for cloning *MSMEG_3743* in pET22b	GGAATTCCATATGCCTGACCAGCGGCGGTC
pET22b-*MSMEG_3743*-RP	RP for cloning *MSMEG_3743* in pET22b	CGG CTCGAG CACGCCGAACCGGTGGATG
MSMEG_3743 K52A-FP	FP for SDM PCR of *MSMEG_3743K52A*	ATGTGCAACCAGGCGGGCGGC
MSMEG_3743 K52A-RP	RP for SDM PCR of *MSMEG_3743K52A*	GCCGACGCCGCCCGCCTGGTTG
MSMEG_3743 K57A-FP	FP for SDM PCR of *MSMEG_3743K57A*	GCGGCGTCGGCGCGACCACGT
MSMEG_3743 K57A-RP	RP for SDM PCR of *MSMEG_3743K57A*	GTCGACGTGGTCGCGCCGACG
pJEX55-*MSMEG_3743*-FP	FP for cloning *MSMEG_3743* in pJEX55	ATGCAGGATCCATGCCTGACCAGCGGCGG
pJEX55-*MSMEG_3743*-RP	RP for cloning *MSMEG_3743* in pJEX55	TGCATGAATTCTCACACGCCGAACCGGTG
pUAB400-*MSMEG_3743*-FP	FP for cloning *MSMEG_3743* in pUAB400	CCGGAATTCGTGGGCCTGACGGGCCGG
pUAB400-*MSMEG_3743*-RP	RP for cloning *MSMEG_3743* in pUAB400	CCCAAGCTTTCACACGCCGAACCGGTGGA
pUAB300- *MSMEG parB*-FP	FP for cloning *MSMEG parB* in pUAB300	CGCGGATCCATGAATCAGCCGGCACGC
pUAB300- *MSMEG parB*-RP	RP for cloning *MSMEG parB* in pUAB300	CCCAAGCTTTTACTCGTTCTGGGCGCT
pUAB400-*Rv1708*-FP	FP for cloning *Rv1708* in pUAB400	GGAATTCTTGCCTGCGGGTCTCCCG
pUAB400-*Rv1708*-RP	RP for cloning *Rv1708* in pUAB400	CCCAAGCTTTCACATGCCAAATCGGTCGATC
pUAB300-*Mtb. parB*-FP	FP for cloning *Mtb. parB* in pUAB300	GGAAGATCTCATGACCCAGCCGTCACG

pUAB300-*Mtb. parB*-RP	RP for cloning *Mtb. parB* in pUAB300	CCCAAGCTTTTACAGAGCGTCCCTGTGC
pUAB300-*Mtb. scpA*-FP	FP for cloning *Mtb. scpA* in pUAB300	CGGGATCCGTGAACGGCCTTCAGAAC
pUAB300-*Mtb. scpA*-RP	RP for cloning *Mtb. scpA* in pUAB300	CCCAAGCTTTCACAAGCGCCGCTCCTT
pUAB300-*Mtb. scpB*-FP	FP for cloning *Mtb. scpB* in pUAB300	CGGGATCCGTGACCGAACATATGCCC
pUAB300-*Mtb. scpB*-RP	RP for cloning *Mtb. scpB* in pUAB300	CCCAAGCTTTCAATCACGGTCCACGTC
pUAB300-*Rv1707*-FP	FP for cloning *Rv1707* in pUAB300	GGAAGATCTGTGTTACAACGAATCGCTAG
pUAB300-*Rv1707*-RP	RP for cloning *Rv1707* in pUAB300	CCCAAGCTTTCAGGCGGATTCGAGGAC
pGEX6P1-*Rv1708*-FP	FP for cloning *Rv1708* in pGEX6P1	GGAATTCTTGCCTGCGGGTCTCCCG
pGEX6P1-*Rv1708*-RP	RP for cloning *Rv1708* in pGEX6P1	CCGCTCGAGTCACATGCCAAATCGGTCGATC
pET22b-*Mtb. scpA*-FP	FP for cloning *Mtb. scpA* in pET22b	GGAATTCCATATGGTGAACGGCCTTCAGAACGA
pET22b-*Mtb. scpA*-RP	RP for cloning *Mtb. scpA* in pET22b	CCGCTCAGCAAGCGCCGCTCCTTCTC
pET22b-*Mtb. parB*-FP	FP for cloning *Mtb. parB* in pET22b	GGAATTCCATATGACCCAGCCGTCACGCAGA
pET22b-*Mtb. parB*-RP	RP for cloning *Mtb. parB* in pET22b	CCCAAGCTTCAGAGCGTCCCTGTGAAG

Table S1: Oligonucleotides used in this study
